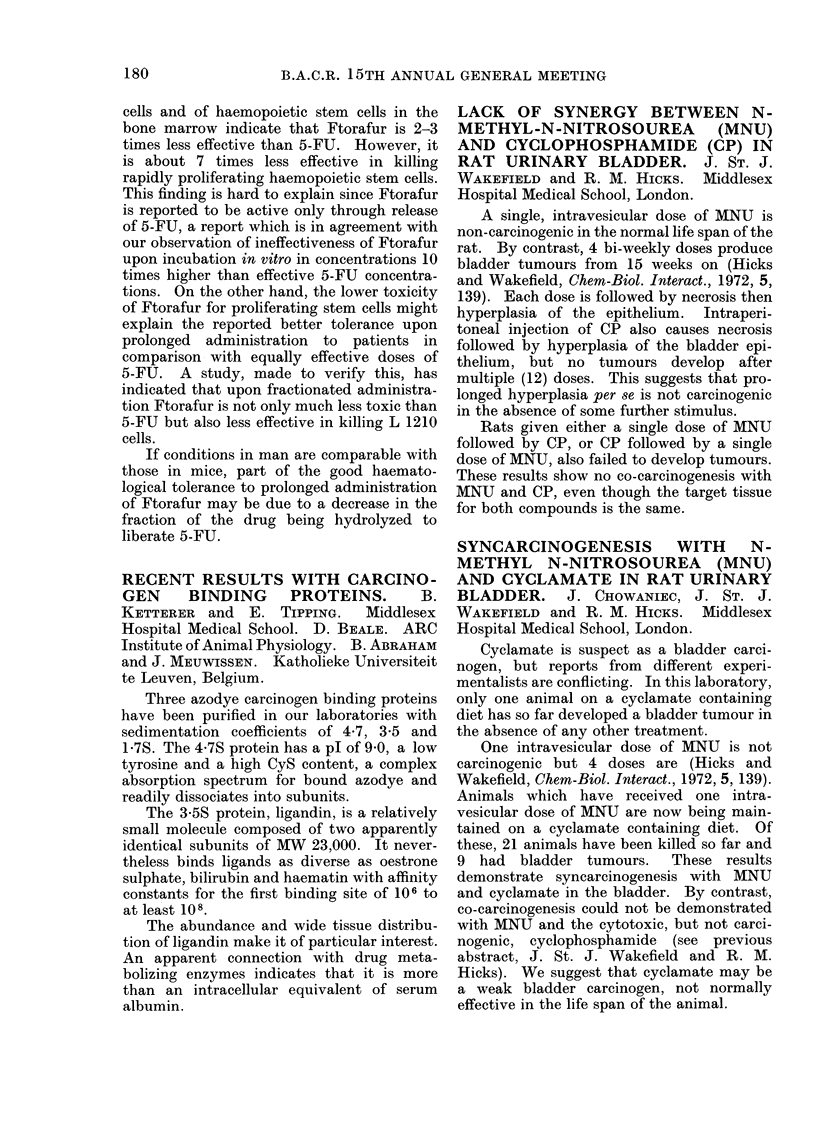# Proceedings: Syncarcinogenesis with n-methyl N-nitrosourea (MNU) and cyclamate in rat urinary bladder.

**DOI:** 10.1038/bjc.1974.157

**Published:** 1974-08

**Authors:** J. Chowaniec, J. S. Wakefield, R. M. Hicks


**SYNCARCINOGENESIS WITH N-METHYL N-NITROSOUREA (MNU) AND CYCLAMATE IN RAT URINARY BLADDER.** J. CHOWANIEC, J. ST. J. WAKEFIELD and R. M. HICKS. Middlesex Hospital Medical School, London.

Cyclamate is suspect as a bladder carcinogen, but reports from different experimentalists are conflicting. In this laboratory, only one animal on a cyclamate containing diet has so far developed a bladder tumour in the absence of any other treatment.

One intravesicular dose of MNU is not carcinogenic but 4 doses are (Hicks and Wakefield, *Chem-Biol. Interact.*, 1972, **5**, 139). Animals which have received one intravesicular dose of MNU are now being maintained on a cyclamate containing diet. Of these, 21 animals have been killed so far and 9 had bladder tumours. These results demonstrate syncarcinogenesis with MNU and cyclamate in the bladder. By contrast, co-carcinogenesis could not be demonstrated with MNU and the cytotoxic, but not carcinogenic, cyclophosphamide (see previous abstract, J. St. J. Wakefield and R. M. Hicks). We suggest that cyclamate may be a weak bladder carcinogen, not normally effective in the life span of the animal.